# Three-dimensional pseudocontinuous arterial spin labeling and susceptibility-weighted imaging associated with clinical progression in amnestic mild cognitive impairment and Alzheimer's disease

**DOI:** 10.1097/MD.0000000000015972

**Published:** 2019-06-07

**Authors:** Qingling Huang, Xuan Cao, Xue Chai, Xiao Wang, Ligang Xu, Chaoyong Xiao

**Affiliations:** a Department of Radiology, Nanjing Medical University Affiliated Nanjing Brain Hospital, Nanjing, China; b Department of Mathematical Sciences, University of Cincinnati, Cincinnati, USA; c Department of Neurology, Nanjing Medical University Affiliated Nanjing Brain Hospital, Nanjing, China.

**Keywords:** 3-dimensional pseudocontinuous arterial spin labeling, Alzheimer's disease, amnestic mild cognitive impairment, susceptibility-weighted imaging

## Abstract

**Background:**

This study aimed to evaluate the value of 3-dimensional pseudocontinuous arterial spin labeling (3D-pcASL) and susceptibility-weighted imaging (SWI) for the early disease-sensitive markers of conversion from amnestic MCI (aMCI) to Alzheimer disease (AD) in this process.

**Methods:**

Forty patients with aMCI and AD respectively were recruited in the study, and 40 healthy subjects were taken as controls. Data were recorded using 3T MR scanner. We assessed the cerebral blood flow (CBF) in 11 different regions of interest, and counted number of microhemorrhages (MB) in 3 regions of brain lobes, bilateral basal ganglia/thalamus, and brain stem/cerebellum, and then investigated correlations between Montreal Cognitive Assessment (MoCA) scores, CBF, and susceptibility-weighted imaging (SWI) features in these 3 groups.

**Results:**

The results revealed that for AD patients, the MoCA scores and CBF values in frontal gray matter (FGM), occipital gray matter (OGM), temporal gray matter (TGM), parietal gray matter (PGM), hippocampus, anterior cingulate cortex (ACC), precuneus, posterior cingulate cortex (PCC), precuneus, basal ganglia and thalamus decreased compared with aMCI patients and control group, and significant difference was revealed among the 3 groups. While in cerebellum, statistical significance was only found between AD patients and control group. On SWI, the average numbers of hemorrhage in regions of lobes for AD patients were significantly higher than aMCI patients and control group. The same results occurred in the bilateral basal ganglia/thalamus. We further found the MoCA score was positively correlated with CBF, but negatively correlated with hypointense signal on SWI.

**Conclusion:**

3D-pCASL and SWI have promising potential to be biomarkers for conversion from aMCI to AD in this process.

## 1 Introduction

Dementia is a collective name for different degenerative brain syndromes, according to Alzheimer's disease (AD) International Association, with the aging of population, the estimated number is about 131.5 million by 2050.^[1]^ AD is the most common cause of dementia. Its symptoms usually begin with a subtle decrease in memory of mild cognitive impairment (MCI), especially in the subtype of amnestic mild cognitive impairment (aMCI).^[2]^ As the condition progressing, these symptoms will gradually deteriorate, including physical, social, emotional, and cognitive processes disturbances, language impairment, and even financial burdens on patients, families, and communities.^[3]^

The pathological hallmarks of AD have been identified, including the deposition of amyloid-β plaques, neurofibrillary tangles, imbalanced metal iron homeostasis, elevated reactive oxygen species, decreasing brain acetylcholine (Ach) levels, even key genes research.^[4–6]^ Early diagnosis and intervention at stage of aMCI can greatly reduce the incidence of AD when symptoms are mild. Therefore, the early diagnosis and intervention treatments of AD are particularly important. There are a variety of diagnostic tools and methods that have been applied to clinical and medical practice. Neuropsychological tests, like Montreal Cognitive Assessment (MoCA), were most commonly used to distinguish aMCI from AD.^[7]^ Cerebro-spinal fluid (CSF) biomarker changes can be used for the preclinical stage of aMCI.^[8]^ Neuroimaging techniques were widely used in the diagnosis of aMCI and AD, including structural and functional magnetic resonance imaging (MRI),^[9]^ and MRI features correlated with neuropsychological findings.^[10]^ Voxel-based morphometry of the structural MRI can be used for the detection of atrophy, in particular the hippocampus which is characteristic for AD.^[11]^ A preliminary study of magnetic resonance spectroscopy found statistically significant lower values of NAA/Cr at the left frontal and left parietal regions in AD compared to aMCI. Meanwhile, the NAA/Cr metabolite ratios of aMCI were much closer to that of AD.^[12]^

MRI perfusion is helpful for differentiating aMCI from AD. Reduced CBF or hypoperfusion is a promising method tightly correlated with the regional consumption of glucose which reflects neuronal activity, and might be as an early marker of neurodegeneration that indicates preceding cognitive decline in AD. Quantification of regional cerebral blood flow (rCBF) of SPECT and PET/CT were recognized as the standard diagnostic procedure of AD that relies on radioactive tracers, but it also restricts wide application in our nation because of its high expense of PET/CT.^[13,14]^ Arterial spin labeling (ASL) MRI utilizes arterial blood water as an endogenous tracer, and provides a sensitive and non-invasive method to detect brain perfusion in AD, even tracks the earliest disease stage of AD progression.^[15]^ A study using 3-dimensional pseudocontinuous arterial spin labeling (3D-pcASL) to predict the severity of AD found that CBF decreased in the precuneus, parietal and occipital lobes in patients with aMCI, and different perfusion in AD group.^[16]^ Some researchers compared CBF with memory scores between aMCI and AD, and found that CBF results were related to memory scores, and discovered relatively increasing values of CBF in the cerebellum, middle orbital frontal lobe, and relatively decreasing blood flow in the hippocampus, temporal cortex, and postcentral gyrus.^[17,18]^ These research findings encourage further investigation of prevention of aMCI and AD.

Microhemorrhages might be a cause of progressive cognitive impairment in the previous research.^[19]^ Susceptibility-weighted imaging (SWI) sequences are much more sensitive for the detection of haemorrhagic than non-contrast CT scan and GE-T2∗ MRI technique.^[20]^ SWI has been widely used in the evaluation of neurodegenerative diseases, vascular malformations, tumors trauma and cerebral amyloid angiopathy. A study using quantified SWI in AD and age-related iron deposition found that iron deposition changes in the globus pallidus can be more easily detected in AD.^[21]^

The primary goal of this study was to investigate the difference of CBF in AD and aMCI patients using 3D-pcASL. We also investigated SWI changes in these 2 groups, then combined that with neuropsychological assessment to investigate if both ASL and SWI could be served as early disease-sensitive markers of aMCI conversion to AD.

## 2 Materials and methods

### 2.1 Participants and assessment

The 30-month cross-sectional prospective study was performed from May 2015 to November 2017, and was approved by our Institutional Review Board. Written informed consent was obtained from all participants. The study covers 40 patients respectively with aMCI and AD (3 cases were excluded from the study because of head movement artifacts), and 40 health controls (HC). These patients including outpatients and inpatients. The diagnosis of aMCI and AD made according the National Institute on Aging-Alzheimer's Association workgroup diagnostic guidelines of 2011 in the study.^[22]^ The 3 groups were matched for gender, age, and education (Table 1 for participant demographic). HCs were hospital staff and community volunteers who were enrolled in the study in our hospital. We conducted MoCA for the 3 groups.^[23]^ They completed MoCA score for routine diagnostic work up, including traditional cognitive domains of memory, attention, executive function, language, and visuospatial ability. Subjects with the following indications were excluded from the study:

1. any syndromes that affect cognitive function such as significant traumatic brain injury, cerebral infarction and local tumor;
2. individuals with a neuropsychiatric disorder, alcohol or drug abuse/dependence;
3. frontotemporal dementia, Lewy body dementia, multiple system atrophy disease or any other medical/psychiatric condition that impact neuropsychological performance.

**Table 1:**
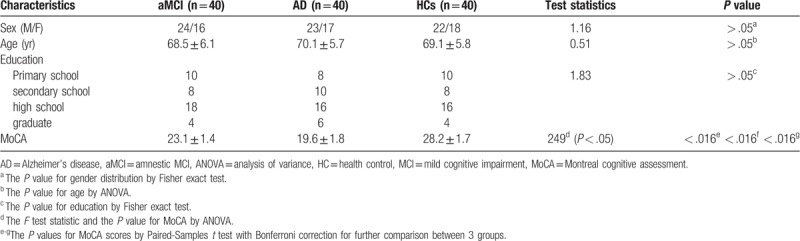
Clinical data and MoCA score of aMCI and AD.

### 2.2 Image acquisition

All participants were examined at 3T (Discovery MR750 system, GE Healthcare, USA) using an 8-channel head coil. Axial 3D T1-weighted structural scan was first acquired. 3D-pcASL perfusion images were collected as follows: TR/TE 6000/21 ms, FOV = 240 mm, matrix = 128 × 128, slice thickness = 4 mm no gap, number of excitations = 3, postlabeling delay = 2000 ms. Imaging parameters of SWI as following: TR/TE = 27/20 ms, FA = 15°, FOV = 230 mm, section thickness = 1.5 mm, matrix = 256 × 256. SWI were reconstructed by correcting phase images and magnitude images. Adjacent magnitude images were post-processed into a minimum intensity projection (MinIP) setting with the slice thickness of 2 mm.

### 2.3 ASL post-processing

**Figure 1:**
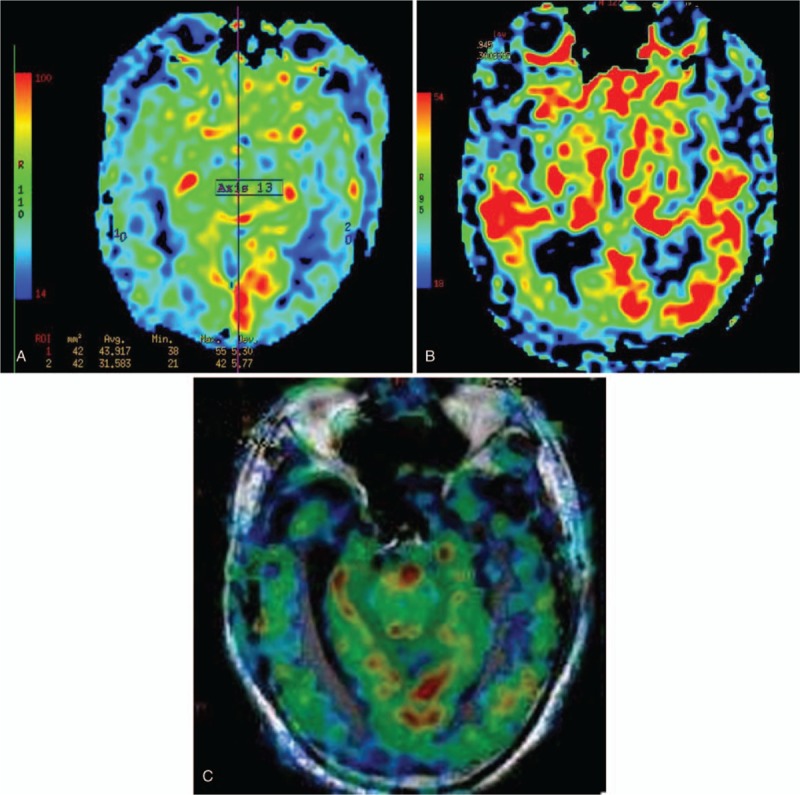
(a–c) Automatically get the quantitative perfusion of the bilateral hemisphere on CBF map using Function Tool software (a). Hypotension on the bilateral TGM in aMCI (b). Color-coded cerebral blood flow map acquired with ASL overlaid on structural T1WI images showed hypoperfusion in the bilateral temporal lobes and occipital lobes in AD (c). AD = Alzheimer's disease, aMCI = amnestic MCI, ASL = arterial spin labeling, CBF = cerebral blood flow, TGM = temporal gray matter.

Data post-processing was performed using GE AW 4.6 workstation Function Tool software for ASL, quantitative perfusion and CBF maps automated generation for each subject, CBF was assessed globally in the entire supratentorial cortex.^[24]^ We selected the bilateral frontal gray matter (FGM), parietal gray matter (PGM), temporal gray matter (TGM), occipital gray matter (OGM) as regions of interest (ROI), ellipses of ROI placement based on T1 structure images which overlaid CBF on T1-weighted structural images (Fig. 1a–c), in order to filter extra-parenchymal signal, avoiding cortical atrophy and partial volume effect, the elliptical ROIs was about 8 to 12 mm^2^, and 3 times in the same area, obtained the average value, and we also measured average CBF values in the bilateral hippocampus, anterior cingulate cortex (ACC), precuneus, posterior cingulate cortex (PCC), thalamus, basal ganglia, and cerebellum.

### 2.4 SWI post-processing

**Figure 2:**
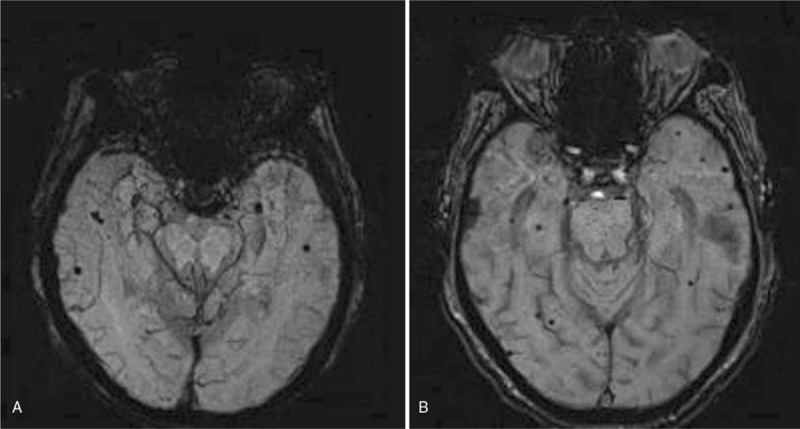
(a-b) There were several microhemorrhages shown on SWI-MinIP in aMCI (a), and more microhemorrhages in AD (b). AD = Alzheimer's disease, aMCI = amnestic MCI.

The number of hemorrhage was counted on SWI. The imaging features analysis included dot-like hypointense signal numbers and location (including brain lobes, bilateral basal ganglia/thalamus, and brain stem/cerebellum), while linear hypointense signal was treated as vessels which would be followed on consecutive SWI images (Fig. 2a-b). All MR images were reviewed by 2 radiologists who were blinded to patient clinical history and outcome.

### 2.5 Statistical analysis

Gender and education difference in the 2 groups of patients (AD and aMCI) and control groups were examined using Fisher exact test. We performed analysis of variance (ANOVA) test for assessing the age difference among 3 groups. For comparing MoCA values, CBF values and numbers of microhemorrhage in different regions among 3 groups, we first conducted ANOVA test for evaluating the overall difference, followed by paired-samples *t* test for comparing MoCA scores, and for assessing difference in CBF and hypointense signals on SWI among the 3 pairs of groups, that is, aMCI vs HCs, AD vs HCs, and aMCI vs AD. For better visualization, we further plotted the scatter plots with regression line and correlation for comparing different MoCA and other characteristic values among 3 groups. We also investigated correlations between MoCA scores, CBF, and SWI. Statistical adjustment for multiple tests (Bonferroni correction) was used for the multivariate analysis. All statistical tests used a significance cutoff of *alpha* = 0.05.

## 3 Results

### 3.1 Sociodemographic and clinical characteristics

**Figure 3:**
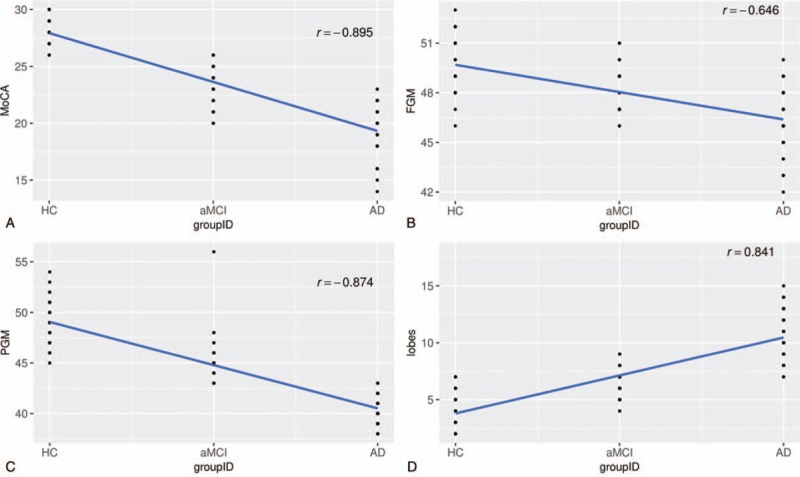
(a–d) Scatter plots with regression line and correlation for comparing different MoCA values (a), FGM values (b), PGM values (c), and average numbers of hemorrhage in regions of lobes (d) among 3 groups. FGM = frontal gray matter, MoCA = Montreal cognitive assessment, PGM = parietal gray matter.

There were no significant differences in gender, age, and education distribution in aMCI and AD and HCs groups (Table 1). However, after conducting ANOVA and pairwise *t* test with Bonferroni correction, significant difference of neuropsychological scores was discovered among 3 pairs of groups, MCI and HC (*P* < .016), AD and HC (*P* < .016), and aMCI and AD (*P* < .016). In particular, the MoCA values for AD patients were significantly lower than that in aMCI and HC groups (Fig. 3a).

### 3.2 CBF measured of aMCI and AD

A controlled CBF study of gray matter in different brain regions of aMCI, AD patients and HCs (Table 2) was conducted. We found that CBF values decreased in FGM, OGM, TGM, PGM, hippocampus, ACC, PCC, precuneus, basal ganglia and thalamus for AD patients compared with aMCI patients and HCs. Meanwhile significant difference was revealed among all 3 groups (Fig. 3b-c). While in cerebellum, statistical significance was only found between AD patients and HCs, but not in the other 2 pairs of groups.

**Table 2:**
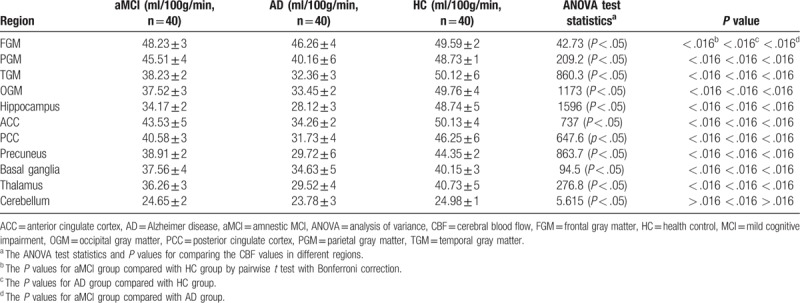
Mean ± standard deviation of CBF in different regions and test results (X ± S).

### 3.3 Hypointense signal on SWI of aMCI and AD

On SWI, dot-like hypointense signals as MBs were found in AD, and also a few hypointense signals were seen in aMCI. The average numbers of hemorrhage in regions of lobes were significantly different in the pairs of aMCI and AD group (*P* < .016), aMCI group and HCs (*P* < .016), and AD and HCs (*P* < .016) (Table 3), where for AD patients, the average numbers of hemorrhage in regions of lobes were significantly higher than the other 2 groups (Fig. 3d). The same results occurred in the bilateral basal ganglia/thalamus, that is, significant difference in the paired group of aMCI and AD group (*P* < .016), aMCI group and HCs (*P* < .016), and AD and HCs (*P* < .016). However, among the 3 groups, there was no significant difference (ANOVA test statistic = 0.408 (*P* > .05)) (Table 3) with respect to the average number of hemorrhage in brain stem/cerebellum. Bonferroni correction for multiple comparisons between MoCA, SWI and CBF were adopted throughout the analysis. In the aMCI and AD groups, the MoCA score was positively correlated with CBF, but negatively correlated with hypointense signal on SWI (corrected *P* = .0136, *P* < .05).

**Table 3:**
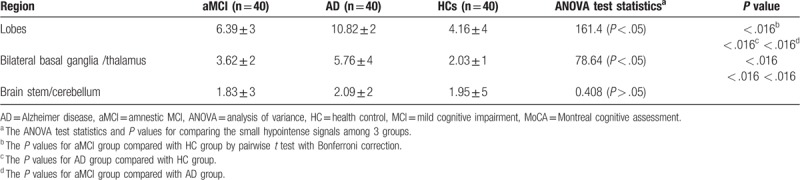
Mean±standard deviation of small hypointense signals in aMCI, AD and HC, and test result s (X ± S).

## 4 Discussion

AD is a neurodegenerative disorder of the nervous system characterized by chronic primary progressive memory and cognitive impairment, aMCI is the early stage of AD that patients only show mild memory loss. Clinical studies have found that about 67% of AD patients are converted from aMCI, while about 75% of aMCI patients eventually develop AD.^[25,26]^ Thus, how to diagnose at aMCI stage is particularly urgent. Clinical diagnosis of AD and aMCI are often based on physical examination, medical history, neuropsychological and radiological examination, laboratory examination, and even pathological examination. As the most influential cognitive assessment tool, MoCA is widely used at home and abroad with good sensitivity, high reliability and validity, and easy operational advantages.^[7,27]^ In this study, neuropsychological tests were performed to assess aMCI and AD. We found that the MoCA score in AD patients was lower than that in aMCI patients, and there was statistical difference among all 3 groups.

Compared with MRI, PET/CT and SPECT are golden standards that can predict conversion from aMCI to AD at different prodromal stages,^[28]^ but it is more expensive, and cannot be widely applied as routine examination in the present medical settings. Therefore, developing and applying sensitive measures for the early diagnosis of aMCI and prevention aMCI to AD conversion seems to be necessary.

Cerebral amyloid angiopathy with MBs is known to be prevalent in patients with AD.^[20]^ Routine MRI examination can detect hemorrhage on DWI. However, the sensitivity and specificity of minimal bleedings at the early stage of AD are not satisfying. SWI is a new technique which can be used to detect the magnetic properties of tissues in recent years, and may be recognized as standard brain imaging in clinical evaluation. By using SWI, MBs might be suggested as a potential imaging marker of AD progression when stable mild cognitive impairment patients compared with MCI progressing patients, during the follow-up, the AD and MCI progressing patients had developed significantly more MBs than HCs and stable mild cognitive impairment patients.^[29]^ On 3T, the resolution can increase from the conventional 1 mm^3^ up to 0.25 mm^3^, and shorten the scanning time of the echo time from 40∼50 ms to 17∼28 ms. In our study, we use 3.0MRI scanner, and found MBs often occur in bilateral hemisphere cortex, subcortex, thalamus/ basal ganglia, and the two groups, aMCI and AD, displayed significant difference. However, less MBs were found in brainstem/cerebellum, and there was no significant difference in aMCI and AD. We also found that iron levels in the temporal cortex (especially hippocampus) were significantly higher than those in HCs, and AD patients showed different levels of iron deposition in bilateral globus pallidus, putamen and hippocampus. A study of autopsy specimens from AD patients on 4.7 T also confirmed the abnormal accumulation of brain iron in bilateral basal ganglia.^[30]^ Although previous study revealed MBs might be related to the Alzheimer's disease process, their presence was not a good candidate for a neuroimaging biomarker in its early phases.^[20]^ In the present study, iron deposition in the bilateral basal ganglia area in AD group was higher than aMCI group, and was consistent with previous studies.^[18,19]^ We also found multiple lobar MBs in the bilateral lobe regions, but in brainstem/cerebellum no significant difference was noted between aMCI and AD group.

Previous studies suggested the vascular-focused theory as AD risk that cerebrovascular damage contributes to cognitive decline.^[31]^ ASL can quantitatively measure the CBF, and 3D-pcASL as the most sensitive ASL technique found weaker CBF differences between HC and aMCI in fronto-parieto-occipital regions than the contrast between HC and AD.^[16]^ In our study, the values of CBF in OGM, TGM, PGM, hippocampus, ACC, PCC, precuneus and thalamus in the aMCI group were significantly higher than that in the AD group. However, no significant difference for the values of CBF in FGM and basal ganglia was observed between the aMCI patients and HCs. Meanwhile, the values of CBF in FGM and basal ganglia were statistically different between HCs and the AD group. These observed lower CBF values in AD than aMCI were most partially agreed with previous studies,^[15,32]^ while in cerebellum, there were no significant differences among the 3 groups, and our finding contradicted the previous study.^[16]^

Reduced CBF means hypoperfusion, may be an early marker of neurodegeneration that initiates a cascade of events preceding cognitive decline in AD.^[33]^ Hypoperfusion in the ACC and PCC may serve as quantitative diagnostic markers for presenile AD and FTD diagnosis in early stage. Currently, CBF did not reveal significant difference in the aMCI group and AD group, but we found significant decrease in the AD group compared to HCs. Several previous ASL studies found that individuals with aMCI exhibited hypoperfusion in parietal cortex, precuneus, PCC and medial temporal lobe, compared with adults with normal cognition or subjective complaints.^[15,16,34]^ In a multi-site analysis from Alzheimer's Disease Neuroimaging Initiative 2, CBF of patients with late aMCI and AD was reduced compared with normal subjects, and lower CBF was associated with disease severity in these patients.^[35]^ Under the resting state, ASL-MRI revealed perfusion changes in basal CBF, these changes of CBF in AD and aMCI are consistent with the vascular hypothesis of AD, and play a crucial role in the pathogenesis development from aMCI to AD.

## 5 Limitation

Although 3D-pCASL has greater signal to noise ratio and reduced specific absorption rate, arterial transient time and motion artifact constitute the most important challenges to the ASL signal. With the advance of ASL perfusion techniques in high or ultra-high MRI, the combination of ASL and SWI has promising potential to be a biomarker for conversion from aMCI to AD. Indeed, the brain characteristics exhibit difference varying with age and race. Chinese brain atlas template will better represent the brain structural difference regardless of age and gender.^[36]^ CBF measure in the manuscript was ROI-based instead of voxel-wised whole brain analysis. In future studies, we will apply Chinese brain atlas to monitor metabolic and structural modalities to detect advanced stages of AD for disease severity, and the treatment response.

## Author contributions

**Conceptualization:** Qingling Huang.

**Data curation:** Qingling Huang, Xue Chai, Xiao Wang, Ligang Xu, Chaoyong Xiao.

**Formal analysis:** Xuan Cao.

**Investigation:** Ligang Xu.

**Project administration:** Qingling Huang.

**Validation:** Xuan Cao, Xiao Wang.

**Visualization:** Chaoyong Xiao.

**Writing – original draft:** Qingling Huang.

**Writing – review & editing:** Xuan Cao, Xue Chai, Xiao Wang, Ligang Xu, Chaoyong Xiao.
